# Monocrystalline Sapphire Stress Field Analysis with Orthorhombic Crystal Orientation Under Vickers Indentation

**DOI:** 10.3390/ma18225136

**Published:** 2025-11-12

**Authors:** Zhongyang Li, Zhaohui Deng, Weiye Yang, Jimin Ge

**Affiliations:** 1 College of Physics and Electronic Engineering, Hainan Normal University, Haikou 571158, China; haha_weiye@163.com; 2 Institute of Manufacturing Engineering, Huaqiao University, Xiamen 361021, China; edeng0080@vip.sina.com; 3 School of Mechanical Engineering, Hunan University of Science Technology, Xiangtan 411201, China; 1030194@hnust.edu.cn

**Keywords:** sapphire, anisotropy, Vickers indentation, crack nucleation and propagation, finite element simulation, stress field analysis

## Abstract

As an irreplaceable optical ceramic material in energy, aviation, and commerce, sapphire is making a further expansion of its application boundaries. Owing to the anisotropy of sapphire, the material properties analysis in the fabrication process is hard but essential. Hence, aiming at investigating the damage behavior of sapphire with different crystal orientations during machining, the nucleation and propagation of cracks in the orthogonal a, c, and m orientations of sapphire under Vickers indentation were explored experimentally and numerically. Firstly, the indentation morphology and indentation cracks of sapphire with different crystal orientations under different loads were studied based on a Vickers indentation tester. In general, the relative errors of the three characteristic parameters, including the half-length of indentation diagonal, the length of crack, and the maximum depth of indentation, are all within 20% between the simulation model and the indentation test results. Then, the nucleation critical loads of different cracks in sapphire under Vickers indentation are determined on the basis of the ceramic materials’ fracture mechanics theory. The critical load value of the median crack of sapphire in both A- and M-planes is less than 0.1 kgf experimentally and simulatively, while C-plane sapphire is between 1 kgf and 2 kgf. Finally, the stress field, displacement–load curve, plastic piling-up height, and dynamic propagation process during Vickers indentation are analyzed, combining the experimental results with a numerical calculation approach.

## 1. Introduction

Sapphire is a typical anisotropic hard-to-machine ceramic material, and its different crystal orientations are used in national defense, consumer electronics, optical windows, and other fields [1,2,3,4,5,6,7]. In recent years, sapphire has been widely applied as a gallium nitride epitaxial substrate material [8,9,10,11,12]. Choosing the proper sapphire with different crystal orientations will affect the luminous efficiency of components. Nevertheless, the difficulty in sapphire fabrication limits its development in multiple applications. The manufacturing process of sapphire mainly undergoes the process chain of grinding, lapping, and polishing. However, due to the extremely high hardness of sapphire, the material removal rate in the lapping and polishing process is inferior, so the damage control of surface and subsurface in the grinding stage will tremendously affect the service performance of sapphire [6,13,14,15,16,17,18].

The plastic deformation mechanism of sapphire dominates the generation and evolution of damage during material removal [19]. When the damage accumulates, the crack caused by plastic deformation gradually changes from stable propagation to unstable propagation, and finally, the sapphire surface fails. Abundant studies indicate that the twin plane and slip plane produced by sapphire in the indentation process are the nucleation sites of the initial microcrack, which belong to the weak interface in the high-energy state [20,21]. Page et al. [22] carried out several indentation tests on C- and R-plane sapphires using the Berkovich indenter. It is found that the indentation creates microcracks under the contact load of only tens of millinewtons, and it is believed that this crack might be the nucleation caused by initial slip, which usually appears on the surface, and only under higher loads. Chan et al. [23] used a Vickers indenter to conduct an indentation test on an A-plane sapphire. Through the transmission electron microscope, not only were the slip and twins on the subsurface of the indentation observed, but the initial microcracks on the twin plane and slip plane were also observed. Haney et al. [24] used a Vickers indenter to conduct a static indentation test on an A-plane sapphire and observed the surface morphology of the indentation. The results show that the direction of the cracks on the indentation surface strongly corresponds with the characteristic angle of the sapphire crystal structure.

The research on the initial stage of induced plastic deformation and crack formation of sapphire benefits from the development of nanoindentation. The “pop-in” phenomenon is a significant discovery based on nanoindentation, and many scholars have carried out research on sapphire nanoindentation [25,26,27,28,29,30]. The Berkovich tip nanoindenter used by Yan et al. [31] was explored on an M-plane single crystal sapphire in response to elastic and plastic factors, and two slip systems were found experimentally and theoretically. Molecular dynamics (MD) simulation further provides a dynamic visualization means for the nanoindentation process [4,32]. Kim et al. [33] applied the MD simulation method for sapphire nanoindentation. It is concluded that the C-plane sapphire formed the rhombohedral twin structures, and the most slip systems were triggered in the R-plane. Lin et al. [34] conducted a similarity simulation with a former researcher and concluded that the R- and A-planes occur in twin/slip systems, which is the prerequisite for C-plane sapphire crack nucleation. Apparently, the existing MD results are an effective supplement to nanoindentation experiments.

After the location of initial crack nucleation is determined, crack propagation is the next focus. The sapphire indentation cracks, including radial crack, median crack, and lateral crack, under the sharp indenter are all sensitive to the crystal orientation. Different from glass and polycrystalline ceramics, sapphire does not crack along the diagonal direction of the indentation under minor load, but along a specific crystal plane [30,35,36]. However, the final crack is determined by the interaction of crystal orientation and load, and the influence of loading rate and fatigue loading cannot be ignored. Luan et al. [37] found that sapphire crystal orientation has a strong impact on the crack propagation by carrying out quasi-static indentation tests on C- and A-plane sapphire using a continuous indentation tester. Wang et al. [38] reached a similar conclusion by utilizing the high-frequency cyclic impact test device and also proposed different crack system models for sapphire with different crystal orientations.

Additionally, considering that the three crystal orientations of a, c, and m in sapphire are mutually orthogonal, many experiments will explore the anisotropy of sapphire by directly investigating the three crystal orientations at the same time, including ultraviolet laser scribing [39], grinding [40,41], etc.

In this paper, with the aim of investigating the damage behavior of sapphire with different crystal orientations during machining, the nucleation and propagation of cracks in the orthogonal a, c, and m orientations of sapphire under Vickers indentation were explored experimentally and numerically. Firstly, the indentation morphology and indentation cracks of sapphire with different crystal orientations under different loads were studied based on a Vickers indentation tester. Then, the nucleation critical loads of different cracks in sapphire under Vickers indentation are determined based on the fracture mechanics theory of ceramic materials. Finally, the stress field, displacement–load curve, plastic piling-up height, and dynamic propagation process during Vickers indentation are analyzed, synthesizing the experimental results and numerical calculation approach.

## 2. Experiments and Simulation

### 2.1. Experiment Set Up

**Figure 1:**
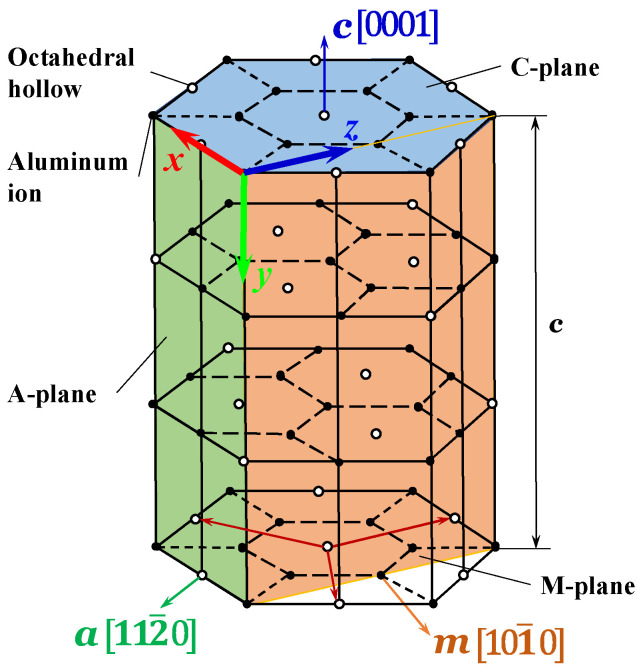
Hexagonal unit cell diagram of sapphire.

The Vickers indentation test utilized sapphire (α-Al_2_O_3_) ceramic blocks produced by Xuzhou Hengsheng Semiconductor Material Factory (Xuzhou, China), which were inspected for the crystal orientation using an orientation instrument (Beijing Shuaiyi Technology Co., Ltd., Beijing, China) after production. Sapphire is a typical difficult-to-machine ceramic material with anisotropic properties. The hexagonal unit cell diagram of sapphire, shown in Figure 1, illustrates that the three crystal orientations a [$11\bar{2}0$], c [0001], and m [$10\bar{1}0$] of sapphire are orthogonal. The Vickers indenter is made of diamond, and the force is transferred to the sapphire surface through the loading process (loading, holding, and unloading).

**Figure 2:**
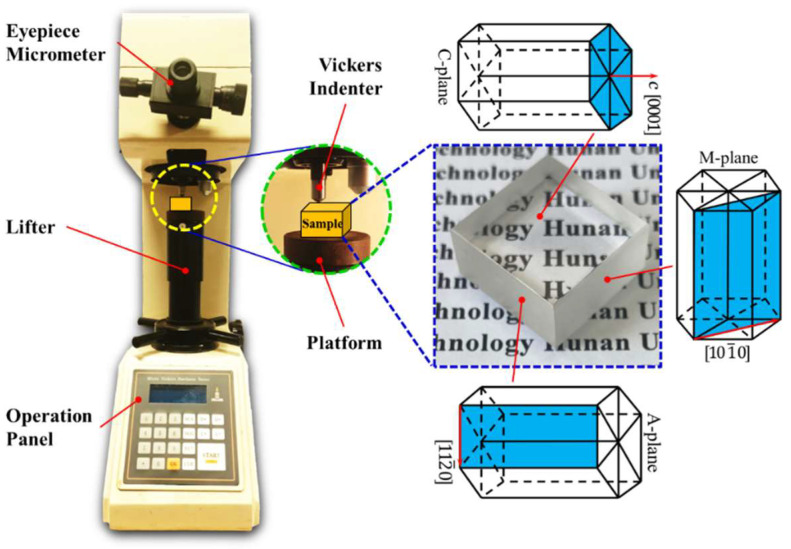
Vickers indentation apparatus and properties of sapphire block.

Sapphire ceramic indentation tests were performed on the Micro Vickers Hardness Tester apparatus (type: HV-1000/HV-1000Z, Shanghai Xinmao Scientific Instrument Co., Ltd., Shanghai, China) at room temperature. The sapphire block undergoes fixturing in a dedicated holder to align its test surface in a perpendicular orientation to the indenter, with a maximum permitted angular deviation of one degree. Upon initiation, the equipment executes an automated sequence: the indenter loads, maintains position for the preset duration, and then unloads. The resulting morphology can then be examined using the integrated high-resolution CCD camera, which is an accessory of the Vickers indentation Hardness Tester apparatus. The diamond indenter is a regular quadrangular pyramid, and the dihedral angle is 136°. The sizes of sapphire ceramics with different crystal orientations are all 20 mm × 20 mm × 10 mm. Among them, the square surface of the sapphire ceramic block is the active surface of the indentation test, so it has been ultra-precisely polished to achieve an ultra-smooth surface with minimum surface damage. The sapphire ceramic block with c-orientation is shown in Figure 2. Hence, the polished surface of the sapphire ceramic block is the C-plane, and the other two matte surfaces are the A- and M-planes that are orthogonal to it. The processing methods of the sapphire ceramic blocks on the a and m orientations are the same as those on the c-orientation. The loads provided by the Vickers indentation apparatus are 1 kgf, 3 kgf, 5 kgf, 10 kgf, 20 kgf, and 30 kgf, respectively, enabling the exploration of the crack propagation of sapphire with different crystal orientations under different loads. The holding time of each indentation test is 10 s. Each sapphire ceramic block was tested on only one polished surface. The polished surface was subjected to three different loads at three suitable positions to avoid mutual interference.

### 2.2. Simulation Modeling

The orthotropic sapphire Vickers indentation finite element simulation model was numerically built by utilizing commercial software ABAQUS (version 2025). Based on the Hill48 anisotropic yield criterion, the plastic deformation of sapphire under Vickers indentation was investigated, and its orthotropic constitutive equation is shown in Equation (1). The mechanical characteristic parameters of orthotropic sapphire and diamond are demonstrated in Table 1.

**Figure 3:**
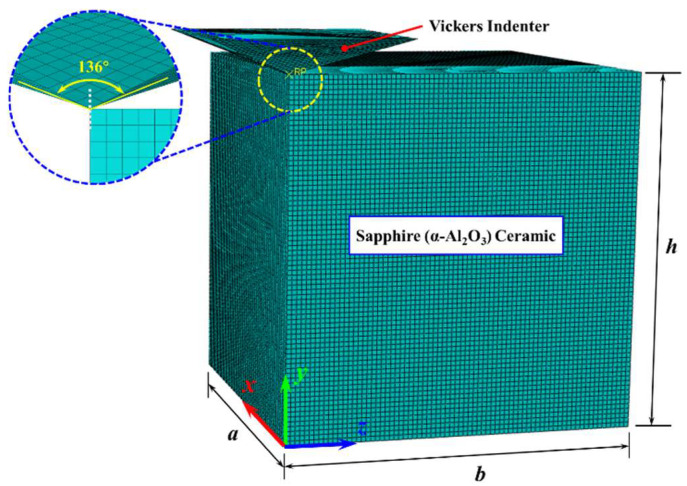
Finite element simulation model.

Taking precedence over the ensuring of computational accuracy and the reduction in the computational memory, the rigid body can be equated to the diamond indenter, owing to the consideration that the mechanical performance of the diamond indenter is much larger than that of sapphire. All simulations in this work employ a consistent base unit system of μm-μg-μs. From this foundation, the density is expressed in 1 × 10^9^ kg/m^3^, stress in GPa, acceleration in 1 × 10^6^ m/s^2^, and force in 1 × 10^−3^ N, with all other physical quantities deriving accordingly. Considering the symmetry of the orthotropic, a quarter sapphire ceramic workpiece was taken as the simulation model, with a size of 300 μm × 300 μm × 300 μm. In addition, the full size of the Vickers indenter simulation model is directly taken without symmetry treatment. The full-size finite element model of the diamond Vickers indenter and a quarter finite element model of sapphire ceramics are illustrated in Figure 3. Given that the indentation process induces significant plastic deformation on the sapphire surface, an adaptive mesh was defined in critical regions. This strategy prevents computational failure due to excessive mesh distortion and ensures the output of precise results. During the Vickers indentation process, the friction between the diamond indenter and the sapphire ceramic will be generated due to mutual contact. In consideration of the limited influence of friction on the final result, the average value f = 0.12 is taken as the friction coefficient input into the model [42,43]. The initial contact point between the diamond indenter and the sapphire is set as the coordinate origin. The coordinate system setting of the finite element simulation model is consistent with the crystal orientation coordinates in Figure 1. The load transferred through the Vickers indenter was applied on the sapphire along the *O*-*y* direction, and the set load was consistent with the experimental value.

(1) $$\left\{\begin{array}{l} \varepsilon_{11}\\ \varepsilon_{22}\\ \varepsilon_{33}\\ \gamma_{12}\\ \gamma_{13}\\ \gamma_{23} \end{array}\right\}=\left[\begin{array}{cccccc} 1/E_{1} & -\nu_{21}/E_{2} & -\nu_{31}/E_{3} & 0 & 0 & 0\\ -\nu_{12}/E_{1} & 1/E_{2} & -\nu_{32}/E_{3} & 0 & 0 & 0\\ -\nu_{13}/E_{1} & -\nu_{23}/E_{2} & 1/E_{3} & 0 & 0 & 0\\ 0 & 0 & 0 & 1/G_{12} & 0 & 0\\ 0 & 0 & 0 & 0 & 1/G_{13} & 0\\ 0 & 0 & 0 & 0 & 0 & 1/G_{23} \end{array}\right]\left\{\begin{array}{l} \sigma_{11}\\ \sigma_{22}\\ \sigma_{33}\\ \sigma_{12}\\ \sigma_{13}\\ \sigma_{23} \end{array}\right\}$$

where $\sigma_{ij}$, as well as $\varepsilon_{ij}$ and $\gamma_{ij}$, represent the stress and strain components, respectively. $E_{1}$, $E_{2}$, and $E_{3}$ represent the elastic modulus in three orthogonal directions, respectively. $G_{12}$, $G_{13}$, and $G_{23}$ represent the shear modulus of deformation on the plane composed of 1–2, 1–3, and 2–3, respectively. $\nu_{ij}$ represents the Poisson’s ratio of the material, and $\nu_{ij}/E_{i}=\nu_{ji}/E_{j}$.

## 3. Results and Discussion

### 3.1. Indentation Surface Morphology

**Figure 4:**
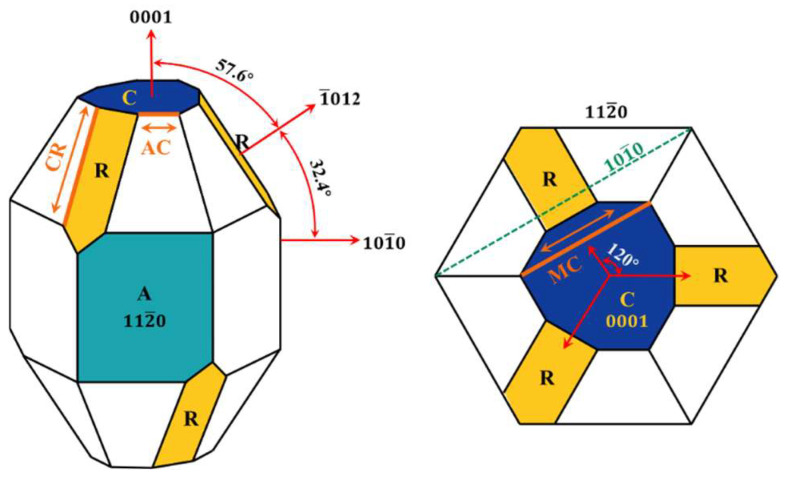
Schematic diagram of the generation of cracks in sapphire.

**Figure 5:**
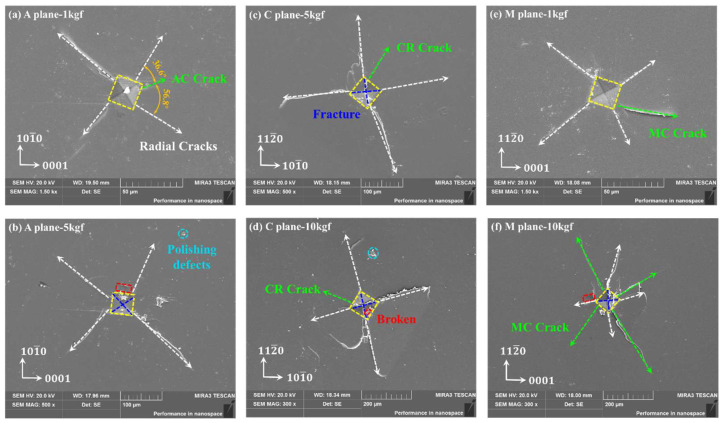
Sapphire Vickers indentation surface morphology with multiple crystal orientations through SEM: (**a**) 1 kgf of A-plane, (**b**) 5 kgf of A-plane, (**c**) 5 kgf of C-plane, (**d**) 10 kgf of C-plane, (**e**) 1 kgf of M-plane, and (**f**) 10 kgf of M-plane.

The Tescan Mira3 LMU field emission scanning electron microscope (SEM) was used to observe and detect the surface indentation morphologies of sapphire with different crystal orientations. Owing to the non-conductive nature of the optical transparency sapphire surface, a conductive gold layer was applied via sputter-coating prior to the examination of the indentation morphology. As illustrated in Figure 4, the sapphire Vickers indentations with multiple crystal orientations are all regular squares with obvious diagonal lines. Along the diagonal at the apex of the square appear radial cracks, while the same cracks also appear not along the diagonal, which are mainly internal cracks in sapphire with anisotropy. As illustrated in Figure 5, different cracks will appear on the sapphire indentation surface with multiple crystal orientations in Vickers indentation: A-plane sapphire generated AC-oriented cracks, C-plane sapphire generated cracks in the CR-direction, and M-plane sapphire generated MC-oriented cracks. These can be found in the Vickers indentation morphology of sapphire in Figure 4. However, cracks have not been reported due to minor loads applied when nanoindentation is used [28,38]. Additionally, a few randomly distributed pits and breaks on the edges of the indentation and on the inner diagonal are generated.

**Figure 6:**
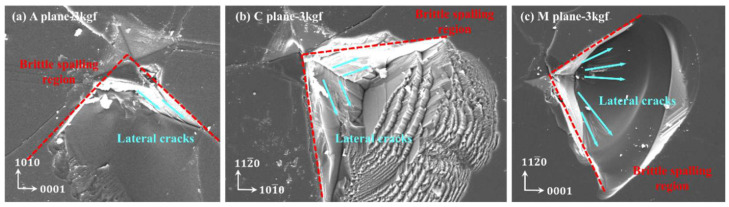
Sapphire Vickers indentation surface morphology under the load of 3 kgf: (**a**) 3 kgf of the A-plane, (**b**) 3 kgf of the C-plane, and (**c**) 3 kgf of the M-plane.

When the load was 3 kgf, material peeling occurred in all three crystal orientations of sapphire, as illustrated in Figure 6. This should be due to unevenness during the loading process. However, through the morphology of the peeling area, it can be seen that due to slight changes in the loading angle, the force in a certain quadrant is biased, and a large number of lateral cracks nucleate and propagate, ultimately resulting in material peeling in this quadrant.

**Figure 7:**
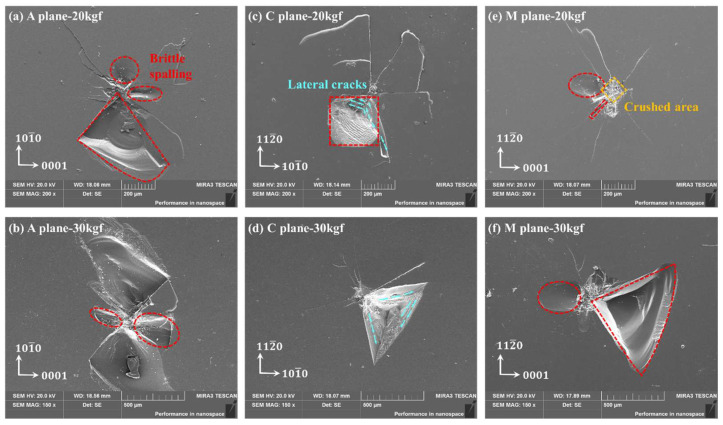
Vickers indentation surface morphology of sapphire under 20 and 30 kgf load by SEM: (**a**) 20 kgf of A-plane, (**b**) 30 kgf of A-plane, (**c**) 20 kgf of C-plane, (**d**) 30 kgf of C-plane, (**e**) 20 kgf of M-plane, (**f**) 30 kgf of M-plane.

Normally, when the load increases, this phenomenon becomes more pronounced, as the nucleation and propagation of lateral cracks are positively correlated with normal loads. As shown in Figure 7, when the load increased to 20 kgf and 30 kgf, all three crystal orientations exhibited large-scale peeling within this quadrant again. The center area of the indentation at 20 kgf and 30 kgf loads is basically crushed, with surface and subsurface properties completely ineffective, while the morphology of the indentation center at 3 kgf is still clear and distinguishable.

### 3.2. Finite Element Simulation Analysis

**Figure 8:**
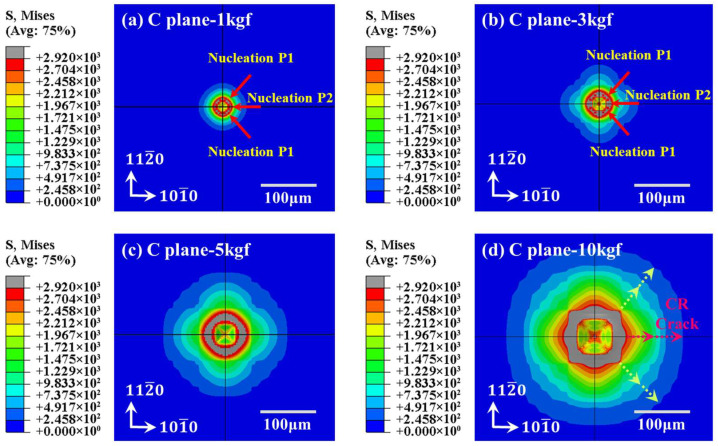
Surface topography simulation of C-plane sapphire under Vickers indentation: (**a**) 1 kgf of C-plane, (**b**) 3 kgf of C-plane, (**c**) 5 kgf of C-plane, (**d**) 10 kgf of C-plane.

Figure 8 is the von Mises stress nephogram of the entire surface of the C-plane sapphire Vickers indentation under different loads. The sapphire surface is indented into a square area by Vickers indentation and varies by load. On the positions of diagonals, boundaries, and vertices, the sapphire yield strength $\sigma_{y}$ is inferior to the value of von Mises stress. And positions with excessive stress values between the two diagonals also existed, which is similar to the indentation phenomenon obtained in Figure 5. Furthermore, both Figure 5 and Figure 8 demonstrated that under the existing parameters, the significant degree of stress concentration and the area of the zone exhibited a positive correlation with the applied load.

**Figure 9:**
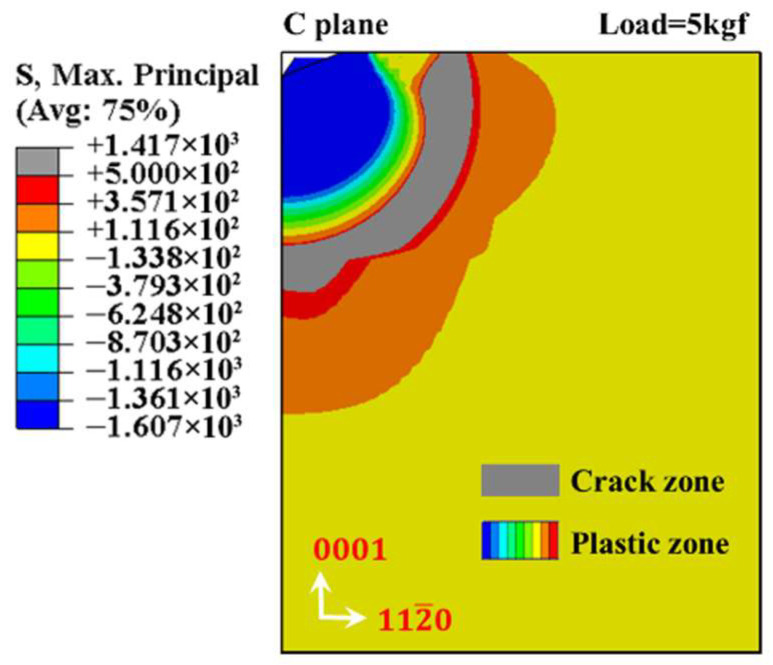
C-plane sapphire with the maximum principal stress nephogram (load 5 kgf).

The validity of the model cannot be accurately characterized simply from the correspondence of indentation morphology. Technically, the correspondence analysis of characteristic parameters between simulation model results and experimental results is more reliable. During the Vickers indentation testing, characteristic parameters, including the half-length of indentation diagonal $a^{m}$, the length of crack $c^{m}$, and the maximum depth of Vickers indentation $h_{l}^{m}$, are the most essential. And the first two can be acquired directly by observing the surface topography, while the third one needs to be obtained indirectly by calculating the trigonometric function of the indenter shape characteristics. For the simulation of indentation morphology, $h_{l}^{s}$ can be obtained directly, while $a^{s}$ and $c^{s}$ need to be identified through stress state analysis. When the unloading process is complete, the half-length of two non-adjacent edges creates a node where the contact stress returns to zero in the original surface can be numerically regarded as $a^{s}$. The crucial impact for crack nucleation is the maximum principal stress, calculated by referencing fracture mechanics theory [44]; that is, when the ultimate stress approaches or surpasses the maximum principal stress, the indentation cracks are generated. Hence, when the unloading process is complete, the length of the indentation center on the original surface to the farthest boundary where the ultimate stress approaches or surpasses the maximum principal stress can be numerically regarded as $c^{s}$. The manufacturer provides the bending strength test values of sapphires in A-, C-, and M-planes, which are 496 MPa, 570 MPa, and 482 MPa, respectively. Considering that the values of the three are relatively consistent, the simulation model in this paper sets the bending strength values of sapphires in three different crystal directions as 500 MPa in order to facilitate comparison. Consequently, the maximum principal stress exceeding 500 MPa can theoretically account for the crack surface inside the sapphire, as demonstrated in Figure 9. Meanwhile, the stress value that is less than zero is regarded as the plastic area being compressed. Due to the finiteness of the model, the internal cracks are not considered.

As demonstrated in Table 2, sapphire indentation characteristic parameters with multiple crystal orientations under different loads are presented. The half-length of indentation diagonal relative errors of a-orientation (1 kgf), c-orientation (3 kgf), and m-orientation (10 kgf) are 4.81%, 6.79%, and 17.94%, respectively, while the crack length relative errors are 2.96%, 6.1%, and 12.3%, respectively, and the maximum indentation depth relative errors are 10.8%, 8.86%, and 5.59%, respectively. In general, the relative errors of the three characteristic parameters are all within 20%, and the simulation model can better correspond to the indentation test results. An important reason for the error is that the simulation model established in this paper only contains three orthogonal crystal orientations of sapphire. However, the actual crystal orientations of sapphire are numerous and not orthogonal.

### 3.3. Judgment of Indentation Cracks

Vickers indentation produces radial cracks, lateral cracks, and half-penny cracks in the material under various loads. However, only the observation of Vickers indentation surface morphology can hardly recognize the accurate type of sapphire indentation cracks under different loads. The driving force of radial crack initiation comes from the residual stress field, which makes the deformation zone become an elastic–plastic “expanding cavity”. The generation of radial cracks is insensitive to the purity of the surface. As a reference to the Lawn–Evans (LE) model [45], a median crack can be generated as the load F approaches or surpasses a numerical criticality. Theoretically, the calculation formulas for the numerical criticality of radial and median cracks are

(2) $$F_{R}^{*}=\Theta \alpha_{0}H{\left(\frac{K_{IC}}{H}\right)}^{4}$$

(3) $$F_{M}^{*}=54.47H\left(\frac{\alpha_{0}}{\eta^{2}\theta^{4}}\right){\left(\frac{K_{IC}}{H}\right)}^{4}$$

where Θ is a dimensionless factor and $\Theta =1/f_{c}^{2}$. $K_{IC}$ is the fracture toughness. H is the Vickers hardness and $H=F/\alpha_{0}\pi a^{2}$.$\alpha_{0}$, η and θ possess the relation to the shape of the indenter ($\alpha_{0}$ = π/2, η = 1 and θ = 0.2 while the Vickers indenter). $F_{R}^{*}$ is the radial crack critical load, and $F_{M}^{*}$ is the median crack critical load. Considering the calculation of Θ is confronted with many uncertainties, Θ will not be carried out in a specific analysis.

As it is commonly a constant, the fracture toughness $K_{IC}$ was applied to realize a description of the ability against the crack propagation or breaking in an unstable state. Many approaches for calculating fracture toughness have emerged, such as the single-sided notched beam (SENB), the double cantilever beam (DCB), the gable notched beam (CNB), and the indentation method (IM). Among them, IM for fracture toughness calculation combining theoretical and empirical means is widely utilized owing to its simple operation and low requirements for sample quality, although there are difficulties in the calculation accuracy. As a reference to the Lawn–Evans–Marshall (LEM) model [46], $K_{IC}$ is obtained through theoretical numerical calculation by

(4) $$K_{IC}=\xi_{V}^{R}{\left(\frac{E}{H}\right)}^{\frac{1}{2}}Fc^{-\frac{3}{2}}$$

where $\xi_{V}^{R}$ is the variable related to the geometry of the indenter, and the Vickers indenter value measured by Anstis et al. is 0.016 ± 0.004 [47]. F is the load, c is the crack length, E is the elastic modulus, and H is the Vickers hardness.

**Figure 10:**
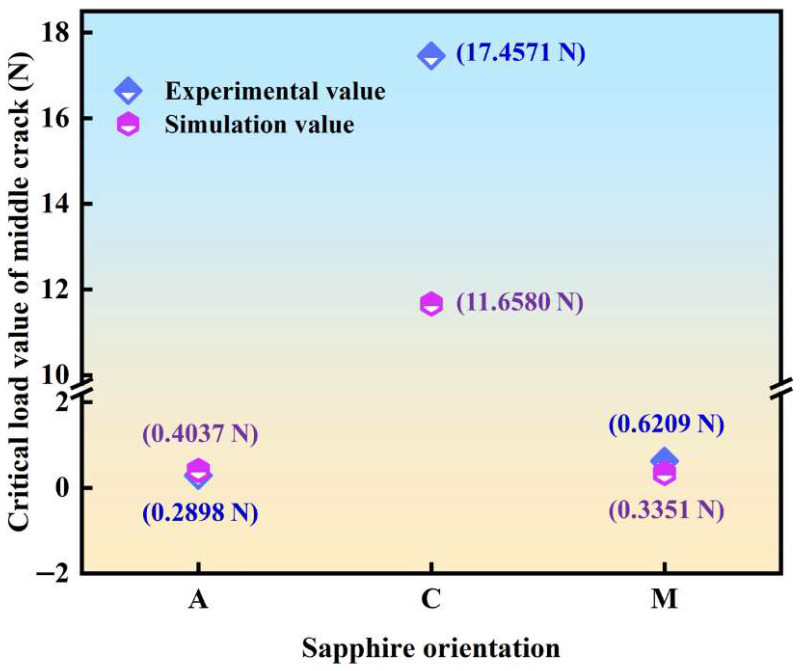
The numerical criticality of sapphire with multiple crystal orientations to produce a median crack.

Experimentally, the average fracture toughness values of a-orientation (1 kgf), c-orientation (3 kgf), and m-orientation (10 kgf) are 2.43 $\mathrm{MPa}\cdot m^{1/2}$, 6.77 $\mathrm{MPa}\cdot m^{1/2}$, and 2.94 $\mathrm{MPa}\cdot m^{1/2}$, with the corresponding average simulation ones being 2.64 $\mathrm{MPa}\cdot m^{1/2}$, 6.12 $\mathrm{MPa}\cdot m^{1/2}$, and 2.52 $\mathrm{MPa}\cdot m^{1/2}$, which are summarized in Table 3. Hence, the critical load of sapphire with different crystal orientations to produce a median crack can be calculated by Equation (3) and is illustrated in Figure 10. The critical load of median crack and the fracture toughness possess a positive correlation. C-plane sapphire has a higher fracture toughness value, so the critical load value of its median crack is greater than that of A- and M-planes. The critical load value of the median crack of sapphire in both A- and M-planes is less than 0.1 kgf experimentally and simulatively. And the critical load value for C-plane sapphire to produce a median crack is between 1 kgf and 2 kgf experimentally and simulatively. Furthermore, the critical load value for generating radial cracks is also less than 0.1 kgf [48]. Hence, a judgment is further concluded that as the load of C-plane sapphire is 1 kgf, the nucleation of radial crack solely occurs while the nucleation of median crack does not. However, under the load set in this paper, the nucleation of both radial and median cracks occurred in A- and M-plane sapphire.

### 3.4. Indentation Cracks Propagation Process

**Figure 11:**
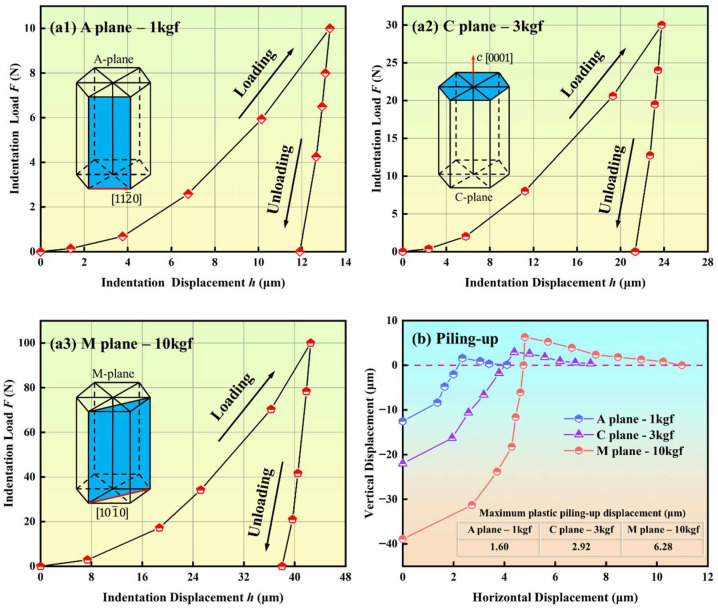
Load–displacement curve of Vickers indentation: (**a1**) A-plane sapphire under 1 kgf load, (**a2**) C-plane sapphire under 3 kgf load, (**a3**) M-plane sapphire under 10 kgf load, (**b**) plastic piling-up displacement curve.

Considering that the experimental observation is the indentation result information, the dynamic indentation process information can be obtained through finite element simulation. In Figure 11, the indentation load–displacement curve, including A-plane sapphire under a 1 kgf load, C-plane sapphire under a 3 kgf load, and M-plane sapphire under a 10 kgf load, is demonstrated. The maximum indentation depth of A-plane sapphire under a 1 kgf load is 13.279 μm, and the linear elastic recovery rate is 10.5%. The maximum indentation depth of C-plane sapphire under a 3 kgf load is 23.775 μm, and the linear elastic recovery rate is 7.42%. The maximum indentation depth of M-plane sapphire under a 10 kgf load is 42.507 μm, and the linear elastic recovery rate is 8.44%. All load–displacement curves in Figure 10 increase from zero to the maximum displacement, and then quickly and elastically recover to the final displacement. The increasing process of the curve corresponds to the loading process of the indentation, and the integral of the curve on the displacement is the work performed by the indenter loading. And the decreasing process of the curve corresponds to the unloading process of the indentation, and the integral of the curve on the displacement is the work performed by the sapphire elastic recovery.

Due to plastic deformation, sapphire Vickers indentation will produce a certain plastic piling-up, which is similar to the plastic core formed on the surface [49]. The plastic piling-up is mainly distributed at the indentation boundary and has a strong relation to the extent of radial crack propagation. In Figure 11, the maximum plastic piling-up displacement of A-plane sapphire under a 1 kgf load is 1.60 μm, the maximum plastic piling-up displacement of C-plane sapphire under a 3 kgf load is 2.92 μm, and the maximum plastic piling-up displacement of M-plane sapphire under a 10 kgf load is 6.28 μm. The maximum plastic piling-up displacement is positively correlated with indentation load and radial crack length.

**Figure 12:**
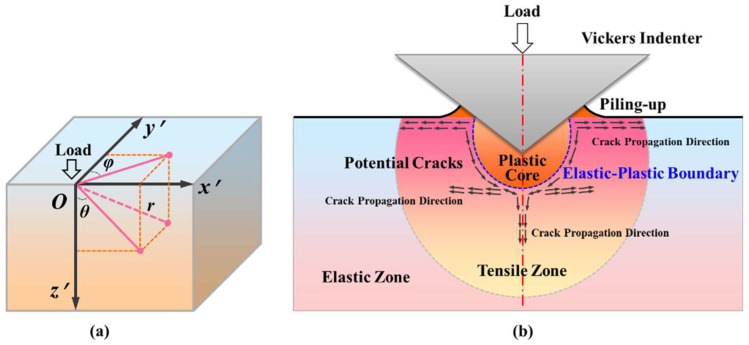
Stress field of sapphire Vickers indentation: (**a**) spherical coordinates and (**b**) indentation stress field and cracks.

A strong high isostatic pressure zone will be generated near the contact point when the Vickers indenter contacts the sapphire surface, resulting in significant local plastic deformation of the sapphire. Therefore, the indentation stress field formed on the sapphire surface is actually a complex elastic–plastic stress field. As shown in Figure 12b, two independent elastic stress fields around the sapphire indentation zone exist:

(i) One is caused by the applied indentation pressing load, which will be entirely recovered after the indentation is completed.
(ii) The other is caused by plastic deformation during indentation. Due to the irreversibility of plastic deformation, this stress field will continue to act on the inside of the sapphire after indentation.

Therefore, the actual stress field inside the sapphire Vickers indentation should be composed of elastic components and residual components. When Poisson’s ratio is 0.28 (C-plane sapphire) and 0.25 [50] (A- and M-plane sapphire), the analytical expressions for the Boussinesq and Blister stress fields in spherical coordinates inside the sapphire Vickers indentation are given as follows:

(5) $$\left\{\begin{array}{l} \sigma_{rr}^{C}=\frac{P}{4\pi r^{2}}\left(0.88-6.88\cos\theta \right)+\frac{B}{r^{3}}\left(18.88\cos^{2}\theta -6.88\right)\\ \sigma_{\theta \theta }^{C}=\frac{P}{4\pi r^{2}}\left(\frac{0.88\cos^{2}\theta }{1+\cos\theta }\right)-\frac{B}{r^{3}}\cdot 0.88\cos^{2}\theta \\ \sigma_{\varphi \varphi }^{C}=\frac{P}{4\pi r^{2}}\cdot 0.88\left(\cos\theta -\frac{1}{1+\cos\theta }\right)+\frac{B}{r^{3}}\cdot 0.88\left(2-3\cos^{2}\theta \right)\\ \sigma_{r\varphi }^{C}=\frac{P}{4\pi r^{2}}\cdot \frac{0.88\sin\theta \cos\theta }{1+\cos\theta }+\frac{B}{r^{3}}\cdot 5.12\sin\theta \cos\theta \end{array}\right.$$

(6) $$\left\{\begin{array}{l} \sigma_{rr}=\frac{P}{4\pi r^{2}}\left(1-7\cos\theta \right)+\frac{B}{r^{3}}\left(19\cos^{2}\theta -7\right)\\ \sigma_{\theta \theta }=\frac{P}{4\pi r^{2}}\left(\frac{\cos^{2}\theta }{1+\cos\theta }\right)-\frac{B}{r^{3}}\cos^{2}\theta \\ \sigma_{\varphi \varphi }=\frac{P}{4\pi r^{2}}\left(\cos\theta -\frac{1}{1+\cos\theta }\right)+\frac{B}{r^{3}}\left(2-3\cos^{2}\theta \right)\\ \sigma_{r\varphi }=\frac{P}{4\pi r^{2}}\cdot \frac{\sin\theta \cos\theta }{1+\cos\theta }+\frac{B}{r^{3}}\cdot 5\sin\theta \cos\theta \end{array}\right.$$

where $\sigma^{C}$ is C-plane sapphire’s actual stress field, while σ is A-/M-plane sapphire’s actual stress field. P is the load of indentation, and r is the length of any point within the field of stress to the indentation center. φ is the included angle between the $Oy^{\prime }$ axis and the projection of the line between any point in the field of stress and the indentation center on the $x^{\prime }Oy^{\prime }$ plane, and θ is the included angle between the $Oz^{\prime }$ axis and the projection of the line between any point in the field of stress and the indentation center on the $x^{\prime }Oz^{\prime }$ plane, which are illustrated in Figure 12a. B, a constant, is the local stress field strength, and B=6EδV/5π, where δV is the indentation volume, and E is the elastic modulus.

Furthermore, the strain source of the elastic half-space free surface derives from the plastic core formed by Vickers indentation, and the maximum tensile stress in this range mostly appears on the elastic–plastic boundary, that is, the nucleation position of the initial crack. Generally, the indentation boundary on the sapphire original surface generates the nucleation of radial cracks, while the elastic–plastic boundary along the loading direction of the indenter generates the nucleation of median cracks and lateral cracks. Therefore, the positions of θ=±π/2rad and θ=0rad in the spherical polar coordinate system need to consider the distribution of the principal stress. Similar to the expression of the actual stress field, when Poisson’s ratio is 0.28 (C-plane sapphire) and 0.25 [50] (A- and M-plane sapphire), the expression of the driving force for nucleation of sapphire Vickers indentation crack is as follows:

(7) $$\left\{\begin{array}{l} \sigma_{R}^{C}=\sigma_{\varphi \varphi }^{C}\left(\theta =\pm \pi /2rad\right)=-0.22p+1.76q\\ \sigma_{M}^{C}=\sigma_{\theta \theta }^{C}\left(\theta =0rad\right)=0.11p-0.88q\\ \sigma_{L}^{C}=\sigma_{rr}^{C}\left(\theta =0rad\right)=-1.5p+12q \end{array}\right.$$

(8) $$\left\{\begin{array}{l} \sigma_{R}=\sigma_{\varphi \varphi }\left(\theta =\pm \pi /2rad\right)=-0.25p+2q\\ \sigma_{M}=\sigma_{\theta \theta }\left(\theta =0rad\right)=0.125p-q\\ \sigma_{L}=\sigma_{rr}\left(\theta =0rad\right)=-1.5p+12q \end{array}\right.$$

where $\sigma^{C}$ and σ are the actual stress fields of C-plane sapphire and A-/M-plane sapphire, respectively. R represents radial cracks, M represents median cracks, and L represents lateral cracks. $p=P/\pi r^{2}$ (in order to eliminate the singularity in the Vickers indentation process, the original concentrated load is uniformly distributed in the entire indentation deformation area is a necessary assumption) and $q=B/r^{3}$.

**Figure 13:**
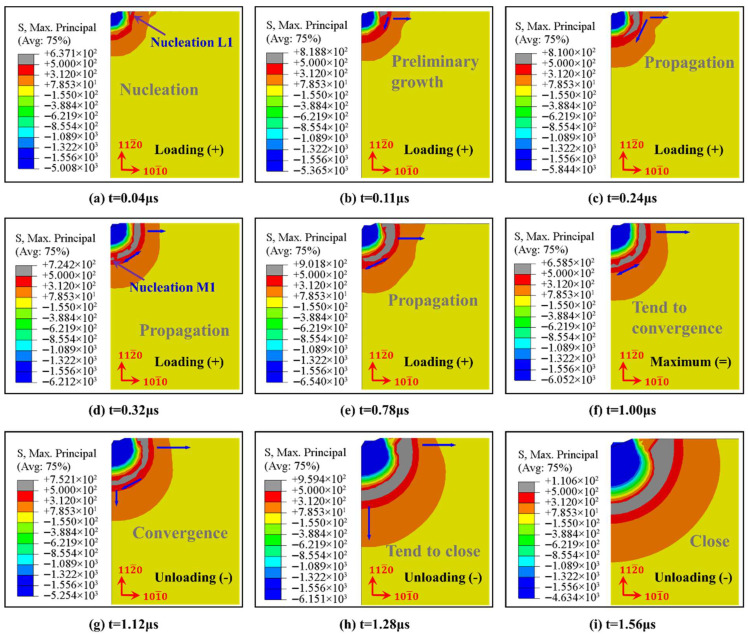
Nucleation and propagation of indentation cracks in Vickers indenter during loading and unloading of A-plane sapphire under a 1 kgf load.

Taking the Vickers indentation process of A-plane sapphire under a 1 kgf load as an example, the whole process is divided into stage loading and stage unloading, as shown in Figure 13.

Stage loading: Equation (8) can reflect the antagonism between the indenter and sapphire in the Vickers indentation process of A-plane sapphire. When loading starts, $\sigma_{R}$ is positive at first, which means that it is when the nucleation of the types of radial crack initially occurs. In Figure 13a–c, the nucleation of the types of radial cracks occurred immediately once the contact took place, and the successive propagation along the horizontal direction (priority at the crystal boundary) and along the elastic–plastic boundary direction was defined. Subsequently, $\sigma_{M}$ is positive, causing the nucleation time of the median crack to be more delayed than that of the radial crack (p<8q), which is consistent with the experimental results of Lankford & Davidson [51]. As the radial crack propagates inside the sapphire along the diagonal of the indentation, however, the radial crack and median crack illustrated in Figure 13d–f gradually tend to converge. Reaching the time of 1.0 μs, the maximum value of both the load of the Vickers indentation and the longitudinal displacement is approached.

**Figure 14:**
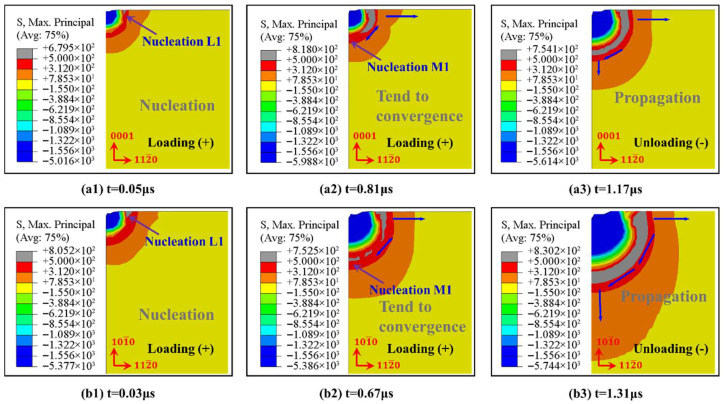
Nucleation and propagation of indentation cracks in Vickers indenter during loading and unloading of C-plane sapphire under 3 kgf load (**a1**–**a3**), M-plane sapphire under 10 kgf load (**b1**–**b3**).

Stage unloading: The consecutive decrease of p during this stage causes $\sigma_{R}$ to increase and $\sigma_{M}$ to decrease gradually. When p=0, $\sigma_{R}$ reaches the maximum, it results in the radial crack having further propagation, and when $\sigma_{M}$ reaches the minimum, it results in the median crack propagating towards the free surface with large tensile stress and converging with the radial crack to generate a system of median/radial cracks, as shown in Figure 13g. When t = 1.56 μs, the cracks are basically closed and will only propagate towards the lowest energy direction, as shown in Figure 13h,i. At the end of the whole unloading process, the elastic recovery of the sapphire terminated, the contact between the indenter and sapphire disappeared, and the propagation of radial and median cracks stopped. A similar process, illustrated in Figure 14, is C-plane sapphire under 3 kgf load and M-plane sapphire under 10 kgf load.

Additionally, the positive and negative values of $\sigma_{L}$ mainly depend on the relative sizes of p and q, thus, the lateral cracks will be generated at the time of unloading as well as even at the end, and then propagate under the drive of the residual stress field [52]. Because it is arduous to make a quantitative judgment, it can only be analyzed by the experimental results to determine whether there is surface breakage. In Figure 5b,d,f, the indentation surface was slightly broken, indicating that lateral cracks were generated.

### 3.5. Stress Field Analysis

The dynamic weakening process of sapphire mechanical performance can be observed through the nucleation and propagation of indentation cracks in the Vickers indenter during loading and unloading, thus providing a solid foundation for subsequent material removal. By reason of the difficulty in observing and measuring, from this perspective, one can seldom obtain focused, effective information revealing the process of crack propagation experimentally.

**Figure 15:**
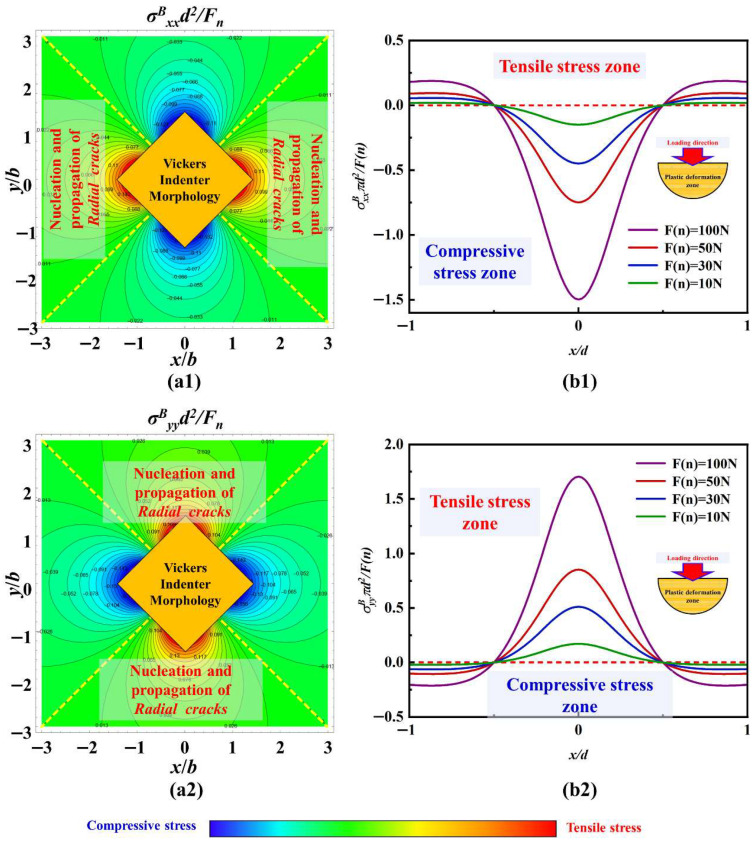
The stress field calculation results with the load of 1 kgf based on the Boussinesq field in the xoy plane: (**a1**) σ^B^_xx_, (**a2**) σ^B^_yy_, (**b1**) the curves of stress value variation with normal load at y/d = 0 of σ^B^_xx_, and (**b2**) the curves of stress value variation with normal load at y/d = 0 of σ^B^_yy_.

The stress field calculation results with the load of 1 kgf are based on the Boussinesq field in the xoy plane under the parameters of c-orientation sapphire, and the curves of stress value variation with normal load at y/d = 0 are shown in Figure 15. In Figure 15(a1,a2), it can be seen that the tensile stress and compressive stress in the principal stress exhibit obvious symmetrical characteristics, and both gradually decrease along the diagonal direction of the indenter. This stress distribution results in the propagation of surface radial cracks under the Vickers indenter, as shown in Figure 5. In Figure 15(b1,b2), the magnitude of normal loading is positively correlated with the change in principal stress. That is to say, when x/d > 1/2, a larger normal load causes greater tensile stress, providing more energy for radial crack nucleation and propagation, despite the amplitude of the overall stress value change being limited. In addition, when x/d < 1/2, an increase in normal load has a significant effect on the stress changes under this condition. As a result, plastic deformation is more likely to occur in this region and quickly transform into brittle fracture. When the normal load is large enough (up to higher than 10 kgf), brittle failure is evident in the middle region; see the experimental results shown in Figure 6.

**Figure 16:**
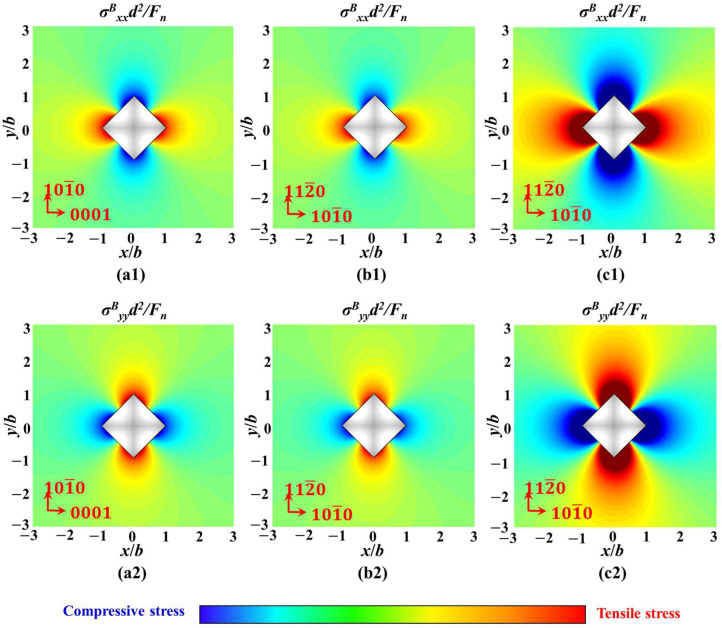
The stress field calculation results based on the Boussinesq field in the xoy plane: (**a1**) the stress values of a-orientation sapphire under a load of 1 kgf of σ^B^_xx_, (**a2**) the stress values of a-orientation sapphire under a load of 1 kgf of σ^B^_yy_, (**b1**) the stress values of c-orientation sapphire under a load of 1 kgf of σ^B^_xx_, (**b2**) the stress values of c-orientation sapphire under a load of 1 kgf of σ^B^_yy_, (**c1**) the stress values of c-orientation sapphire under a load of 10 kgf of σ^B^_xx_, (**c2**) the stress values of c-orientation sapphire under a load of 10 kgf of σ^B^_yy_.

Furthermore, the stress distribution of different crystal orientations in the xoy plane under the same load was also discussed. Figure 16(a1,a2) shows the stress values of a-orientation sapphire under a load of 1 kgf, while Figure 16(b1,b2) shows the stress values of c-orientation sapphire under the same load. Under the same load, the maximum principal stress on the xoy plane of sapphire with different crystal orientations is negatively correlated with Poisson’s ratio. That is to say, under the same load, the constraint stress that c-orientation sapphire needs to overcome will be smaller than that of a-orientation sapphire, thus potentially expanding into longer radial cracks. However, due to the crystal structure of the sapphire material being more conducive to propagating the radial crack of the A-plane, the length of the crack between the two is with an inapparent difference, as shown in Figure 5b,c. The stress distribution of the same crystal orientation in the xoy plane under different loads was also compared. Figure 16(c1,c2) shows the stress values of c-orientation sapphire under a load of 10 kgf. Unlike the difference in crystal orientation, increasing the normal load results in a more significant change in stress. The change in principal stress gradient along the diagonal direction of the indenter is more pronounced, making radial crack propagation more powerful, which is illustrated in Figure 5.

**Figure 17:**
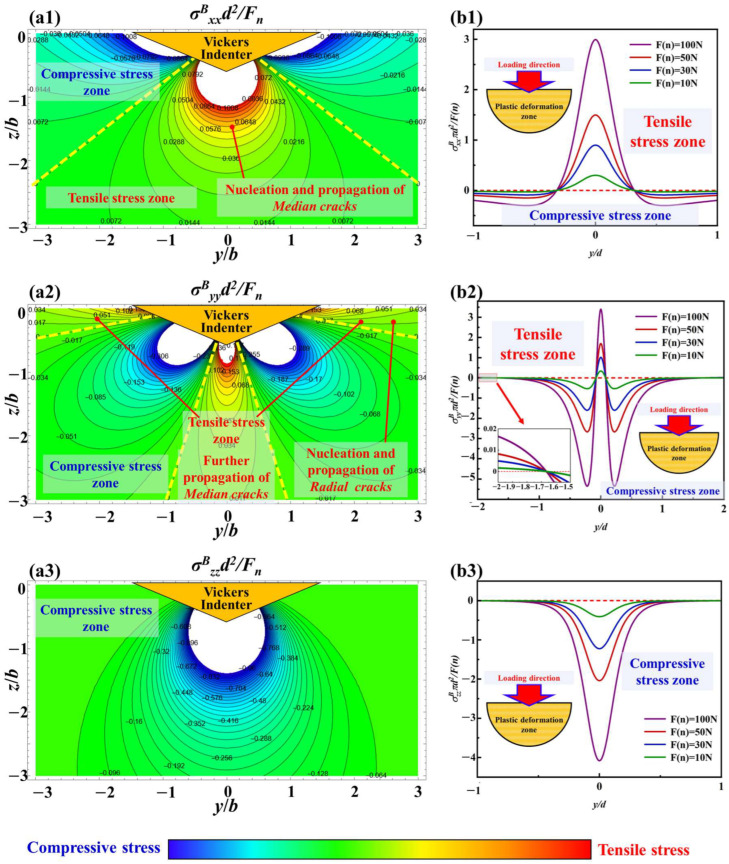
The stress field calculation results with the load of 1 kgf based on Boussinesq field in the yoz plane, (**a1**) σ^B^_xx_, (**a2**) σ^B^_yy_, (**a3**) σ^B^_zz_, (**b1**) the curves of stress value variation with normal load at x/d = 0 of σ^B^_xx_, (**b2**) the curves of stress value variation with normal load at x/d = 0 of σ^B^_yy_, (**b3**) the curves of stress value variation with normal load at x/d = 0 of σ^B^_zz_.

The stress field calculation results with the load of 1 kgf based on the Boussinesq field in the yoz plane under the parameters of c-orientation sapphire, and the curves of stress value variation with normal load at x/d = 0, are shown in Figure 17. In Figure 17(a1,a2), along the loading direction of the Vickers indenter, both σ^B^_xx_ and σ^B^_yy_ have obvious tensile stress regions, which are the areas where median cracks nucleate and propagate. The curves in Figure 17(b1,b2) indicate that as the normal load increases, the tensile stress values for nucleation and propagation of median cracks will also increase with the increase of σ^B^_xx_ and σ^B^_yy_. Therefore, a larger normal load will make it easier for median cracks to nucleate and propagate, or in other words, the time for median crack nucleation will be shorter under a larger normal load. In Figure 17(a2,b2), there is also a tensile stress region near the z = 0 surface, which provides energy for the propagation of radial cracks, making them not only expand horizontally but also have a downward trend.

However, in Figure 17(a3,b3), σ^B^_zz_ only contains compressive stress and shows a positive correlation with the variation in normal load. Excessive compressive stress can suppress the propagation of cracks, especially median cracks along the loading direction.

**Figure 18:**
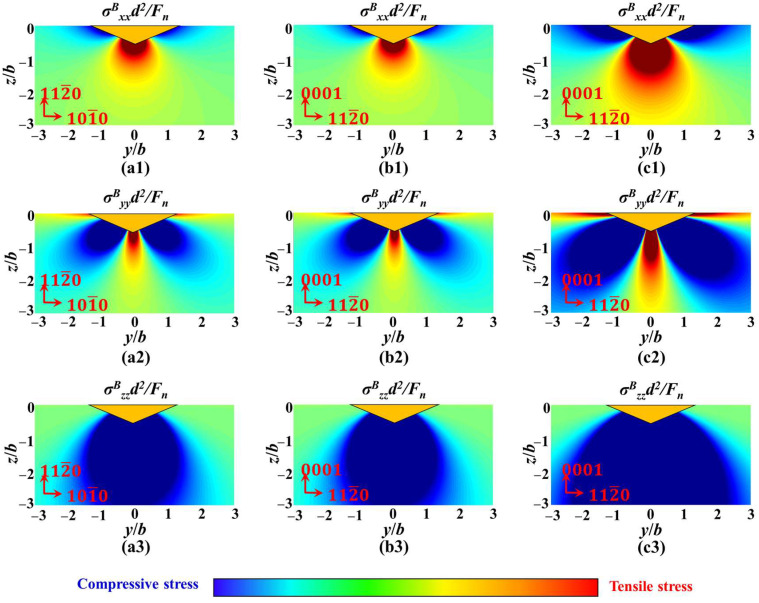
The stress field calculation results based on Boussinesq field in the yoz plane, (**a1**) the stress values of a-orientation sapphire under a load of 1 kgf of σ^B^_xx_, (**a2**) the stress values of a-orientation sapphire under a load of 1 kgf of σ^B^_yy_, (**a3**) the stress values of a-orientation sapphire under a load of 1kgf of σ^B^_zz_, (**b1**) the stress values of c-orientation sapphire under a load of 1 kgf of σ^B^_xx_, (**b2**) the stress values of c-orientation sapphire under a load of 1 kgf of σ^B^_yy_, (**b3**) the stress values of c-orientation sapphire under a load of 1 kgf of σ^B^_zz_, (**c1**) the stress values of c-orientation sapphire under a load of 10 kgf of σ^B^_xx_, (**c2**) the stress values of c-orientation sapphire under a load of 10 kgf of σ^B^_yy_, (**c3**) the stress values of c-orientation sapphire under a load of 10 kgf of σ^B^_zz_.

The parameters in Figure 18 are consistent with the crystal orientation and load in Figure 16. Due to the difference in Poisson’s ratio, the tensile stress along the loading direction and perpendicular to the loading direction of sapphire in a-orientation is greater than that in c-orientation under the same load, as illustrated in Figure 18(a1–a3,b1–b3). Meanwhile, σ^B^_zz_ is insensitive to Poisson’s ratio; therefore, its inhibitory effect on median cracks is similar under the same load. Hence, sapphire in a-orientation is not only more prone to nucleation and propagation of median cracks but also promotes further propagation of radial cracks. Furthermore, under the same direction, an increase in normal load will cause an increase in all principal stresses, thus promoting both median and radial cracks, as illustrated in Figure 18(b1–b3,c1–c3).

**Figure 19:**
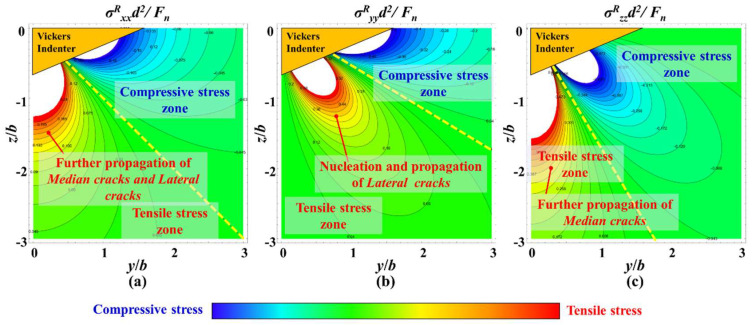
Residual stress field during Vickers indentation unloading process, (**a**) σ^R^_xx_, (**b**) σ^R^_yy_, (**c**) σ^R^_zz_.

During the unloading process of Vickers indentation, the residual stress Blister field will dominate the propagation of cracks. In Figure 19a,c, the residual stress field directly below the Vickers indenter is tensile stress. Therefore, during the unloading process, this tensile stress will further provide energy for the propagation of the median crack. In addition, based on Figure 19a,b, lateral crack nucleation and propagation will also occur along the edge of the plastic deformation zone, which is mainly dominated by σ^R^_yy_. The propagation of lateral cracks will further merge with the radial cracks that propagate downward during the loading process, forming a typical radial-median crack and lateral crack system.

## 4. Conclusions

In this paper, aiming at investigating the damage behavior of sapphire with different crystal orientations during machining, the nucleation and propagation of cracks in the orthogonal a, c, and m orientations of sapphire under Vickers indentation were explored experimentally and in a simulated manner. The indentation morphology and indentation cracks, the nucleation critical loads of different cracks, the stress field, displacement–load curve, plastic piling-up height, and dynamic propagation process during Vickers indentation are analyzed utilizing the experimental results and numerical calculation approach. Relevant conclusions can be summarized as follows.

(1) From experimental results, A-plane sapphire generated AC-orientation cracks, C-plane sapphire generated CR-direction cracks, and M-plane sapphire generated MC-orientation cracks. The indentation diagonal half-length relative errors of a-orientation (1 kgf), c-orientation (3 kgf), and m-orientation (10 kgf) are 4.81%, 6.79% and 17.94%, respectively; the crack length relative errors are 2.96%, 6.1%, and 12.3% and the maximum indentation depth relative errors are 10.8%, 8.86% and 5.59%.
(2) The critical load value of the median crack of sapphire in both A- and M-planes is less than 0.1 kgf experimentally and simulatively, while C-plane sapphire is between 1 kgf and 2 kgf. Hence, as the load of C-plane sapphire is 1 kgf, the nucleation of radial crack occurred slowly, while the nucleation of median crack did not. However, the nucleation of both radial and median cracks occurred in A- and M-plane sapphire under the load of this paper’s experiment.
(3) From simulation results, the maximum depth of Vickers indentation of a-orientation (1 kgf), c-orientation (3 kgf), and m-orientation (10 kgf) are 13.279 μm, 23.775 μm, and 42.507 μm, respectively, and the corresponding linear elastic recovery rates are 10.5%, 7.42%, and 8.44%. The maximum plastic piling-up displacement of a-orientation (1 kgf), c-orientation (3 kgf), and m-orientation (10 kgf) are 1.60 μm, 2.92 μm, and 6.28 μm.
(4) The radial cracks, which commenced earlier than the median crack, nucleate immediately at the initial contact, and then begin to propagate along the horizontal direction (priority at the crystal boundary) and along the elastic–plastic boundary direction. In the unloading stage, the radial crack has further propagation, and the median crack will propagate towards the free surface with large tensile stress and converge with the radial crack to form a median/radial crack system. The generation of lateral cracks will occur during the process of unloading, even at the end. Under the load of 20 kgf, the surface of Vickers indentations with three crystal orientations appeared brittle spalling or even cracking, owing to abundant lateral cracks, nucleated and propagated.

## Figures and Tables

**Table 1:** Mechanical characteristic parameters of orthotropic sapphire and diamond.

Materials	Density *ρ* (g/cm^3^)	Elastic Modulus *E* (MPa)	Yield Strength σ (MPa)	Shear Modulus *γ* (MPa)	Poisson’s Ratio *ν*
Sapphire-c [0001]	3.98	456,490	2920	140,000	0.28
Sapphire-a $\left[11\bar{2}0\right]$	3.98	431,240	2920	140,000	0.25
Sapphire-m $\left[10\bar{1}0\right]$	3.98	411,570	2920	140,000	0.25
Diamond	3.5	1,140,000	-	-	0.07

**Table 2:** Sapphire indentation characteristic parameters with different crystal orientations.

**Crystal** **Orientations**	**Loads (kgf)**	Experimental Value			Simulation Value		
		$a^{m}$ (μm)	$c^{m}$ (μm)	$h_{l}^{m}$ (μm)	$a^{s}$ (μm)	$c^{s}$ (μm)	$h_{l}^{s}$ (μm)
a [$11\bar{2}0]$	1	15.36	60.71	11.32	14.62	58.91	11.89
c [0001]	3	32.54	123.14	24.15	34.75	115.63	22.01
m [$10\bar{1}0]$	10	97.76	187.69	39.86	115.3	210.78	38.92

**Table 3:** Fracture toughness results of experiment and simulation.

	A-Plane	C-Plane	M-Plane
Experiment $K_{IC}$ ($\mathrm{MPa}\cdot m^{1/2}$)	2.43	6.77	2.94
Simulation $K_{IC}$ ($\mathrm{MPa}\cdot m^{1/2}$)	2.64	6.12	2.52

## Data Availability

The original contributions presented in this study are included in the article. Further inquiries can be directed to the corresponding author.
